# Visualizing Psychological Networks: A Tutorial in R

**DOI:** 10.3389/fpsyg.2018.01742

**Published:** 2018-09-19

**Authors:** Payton J. Jones, Patrick Mair, Richard J. McNally

**Affiliations:** Department of Psychology, Harvard University, Cambridge, MA, United States

**Keywords:** network analysis, network psychometrics, psychopathology, multidimensional scaling, graph theory

## Abstract

Networks have emerged as a popular method for studying mental disorders. Psychopathology networks consist of aspects (e.g., symptoms) of mental disorders (nodes) and the connections between those aspects (edges). Unfortunately, the visual presentation of networks can occasionally be misleading. For instance, researchers may be tempted to conclude that nodes that appear close together are highly related, and that nodes that are far apart are less related. Yet this is not always the case. In networks plotted with force-directed algorithms, the most popular approach, the spatial arrangement of nodes is not easily interpretable. However, other plotting approaches can render node positioning interpretable. We provide a brief tutorial on several methods including multidimensional scaling, principal components plotting, and eigenmodel networks. We compare the strengths and weaknesses of each method, noting how to properly interpret each type of plotting approach.

Psychologists have witnessed an explosion of research utilizing network analysis to measure psychological constructs (see Fried et al., 2017 for a review). Networks, which consist of nodes connected to each other by edges, are a useful tool for visualizing and interpreting relational data. Diverse statistical procedures can be applied to analyze network structures. For example, researchers can determine which nodes are most highly connected or whether the network clusters into separate communities of nodes.

Unlike social networks where one directly observes connections between individuals (e.g., friends, enemies; Burt et al., 2013), the edges in psychological networks require statistical estimation, often partial correlations reflecting the strength of association between nodes. In visualizations, green (or blue) edges represent positive associations, and red edges represent negative associations. The thickness of an edge corresponds to the strength of association. Dubbed “network psychometrics,” (Epskamp et al., 2016; Fried et al., 2017), this approach has stimulated many studies estimating networks of various psychological constructs.

In contrast to traditional approaches to psychopathology that regard symptoms as reflecting the presence of a latent disease entity that causes their emergence and covariance, network researchers view mental disorders as emerging from interactions among symptoms (Cramer et al., 2010; Borsboom and Cramer, 2013; Borsboom, 2017). Researchers have therefore endeavored to model disorders as causal systems. Theory motivating this type of analysis posits that mental disorders are phenomena emerging from the causal associations between biological, social, and affective components (Jones et al., 2017).

However, network analysis has not been confined to abnormal psychology. Researchers have applied network analysis in studies on personality (Cramer et al., 2012; Costantini et al., 2015a,b, 2017) and attitudes (Dalege et al., 2016), arguing that traits and attitudes may be better represented as emergent properties of complex networks rather than as underlying latent variables (e.g., dimensional personality factors). Indeed, as this approach becomes more widely known, it is likely that many more psychological constructs will soon be characterized as emergent properties of complex networks (e.g., Barabási, 2011). Thus, understanding the nuances of network analysis is of growing importance in psychology.

In this article, we explore several methods for visualizing networks. Each has advantages and disadvantages. Some foster intuitive spatial interpretation of network structure, whereas others provide little spatial information, but facilitate clarity and aesthetics of network edges. Our tutorial applies exclusively to network visualization; network computation procedures such as node centrality remain identical regardless of the visualization method one uses. We provide brief, simple explanations and examples suitable for psychological researchers who plan to use or interpret network analyses. As this article is not an advanced statistical tutorial, we relegate formulas and other detailed information to an Data Sheet 1 (Appendix). We provide accompanying R code (R Core Team, 2018) in the text throughout this tutorial (Data Sheet 3).

## Visual (Mis)interpretation of networks

Networks enable the visualization of complex, multidimensional data as well as provide diverse statistical indices for interpreting the resultant graphs (e.g., McNally, 2016; Haslbeck and Waldorp, 2017; Jones, 2017; van Borkulo et al., 2017). However, depending on how the network is plotted, visual interpretation of the position of nodes can easily lead one astray. Four misunderstandings about the spatial placement of nodes are common.

First, researchers may assume that the graphical spacing of two connected nodes signifies the magnitude of their association. This is not always true. Depending on the plotting method, two strongly associated nodes may appear far apart, whereas two weakly associated nodes may appear close together.

Second, researchers may mistakenly assume that a node's placement along the X and Y axes signifies a meaningful position on a coordinate plane. For example, consider a network in which OCD symptoms cluster on the right and depression symptoms cluster on the left. A researcher might erroneously conclude that the depression symptoms nearer to the right are “more OCD-like” than those toward the left. The X and Y position of nodes cannot always be interpreted in this way; position of nodes does not necessarily correspond to a meaningful coordinate plane.

Third, researchers may erroneously conclude that a node positioned in the center of the network is a *central* node. Node centrality metrics measure the “importance” of a node in a network, not its physical position in the graph. For example, strength centrality reflects the number and magnitude of connections a node has to other nodes in the network. A node with many strong connections may appear anywhere in the graph, not necessarily in its center. Conversely, nodes appearing near the center of a graph need not be highly central in the network.

Fourth, researchers may incorrectly assume that a network study failed to replicate because the network in the new study appears dramatically different than the original one. Not all plotting methods are stable, and some can be rotated arbitrarily. This can lead to networks that appear wildly different, even though their statistical structures are similar.

Depending on the visualization method, any or all of these assumptions may be incorrect. Researchers can minimize misinterpretations by careful choice of visualization methods and raising awareness about how to interpret each type of visualization accurately.

## Two practical example datasets

In order to demonstrate different types of visualizations, we will use two example datasets from the literature. Both datasets contain information on symptoms of obsessive-compulsive disorder (OCD) and depression. OCD and depression are frequently comorbid (Millet et al., 2004). Moreover, comorbid depression is associated with aggravated OCD symptoms and higher rates of suicide (Torres et al., 2011; Brown et al., 2015). Understanding the complex relationships among OCD and depression symptoms may provide valuable insight for clinicians and researchers.

McNally et al. (2017) used network analysis to examine OCD and depression symptoms in adults. A dataset of these symptoms in 408 adults is available in the MPsychoR package (Mair, 2018). The 26 symptoms were recorded using Likert style self-report scales (Y-BOCS, QIDS-SR; see McNally et al., 2017 for details). Let's load the data (Data Sheet 2).


library("MPsychoR")
data(Rogers)
dim(Rogers)
[1] 408 26


Jones et al. (2018) replicated this analysis in a smaller sample of adolescents. This dataset is also included in the *MPsychoR* package (Mair, 2018). This replication dataset of 87 adolescents provides an opportunity to compare and contrast visualizations with the sample from McNally et al. (2017).


data(Rogers_Adolescent)
dim(Rogers_Adolescent)
[1] 87 26


To preserve space in network visualizations, we will assign a number to each variable for the labels. Numbering and variable descriptions can be found in Table 1.


colnames(Rogers)
    <- colnames(Rogers_Adolescent)<- 1:26


**Table 1:** Nodes in Adult and Adolescent OCD & Depression Networks.

**Number**	**Symptom (Depression)**	**Number**	**Symptom (OCD)**
1	Sleep-onset insomnia	17	Time consumed by obsessions
2	Middle insomnia	18	Interference due to obsessions
3	Early morning awakening	19	Distress caused by obsessions
4	Hypersomnia	20	Difficulty resisting obsessions
5	Sadness	21	Difficulty controlling obsessions
6	Decreased appetite	22	Time consumed by compulsions
7	Increased appetite	23	Interference due to compulsions
8	Weight loss	24	Distress caused by compulsions
9	Weight gain	25	Difficulty resisting compulsions
10	Concentration impairment	26	Difficulty controlling compulsions
11	Guilt and self-blame		
12	Suicidal thoughts, plans, or attempts		
13	Anhedonia		
14	Fatigue		
15	Psychomotor retardation		
16	Agitation		

## Force-directed algorithms (e.g., Fruchterman-Reingold)

Most network studies in psychopathology have used the Fruchterman-Reingold (FR) algorithm to plot graphs (Fruchterman and Reingold, 1991). The FR algorithm is a force-directed graph method (see also Kamada and Kawai, 1989) akin to creating a physical system of balls connected by elastic strings. An elastic string connecting two nodes pulls them closer together, while other nodes draw them apart in other directions. This results in a visually appealing graph where nodes generally do not overlap and edges have approximately the same length.

The aim of force-directed algorithms is to provide aesthetically pleasing graphs by minimizing the number of crossing edges and by positioning nodes so that edges have approximately equal length. Importantly, the purpose of plotting with a force-directed algorithm is *not* to place the nodes in meaningful positions in space. Rather, the intent is to position nodes in a manner that allows for easy viewing of the network edges and clustering structures.

When plotting with the FR algorithm or another force-directed method, one must refrain from making any spatial interpretation. Erroneous interpretations based on spatial arrangement are a common trap as it is difficult to ignore space in a visualization.

**Figure 1:**
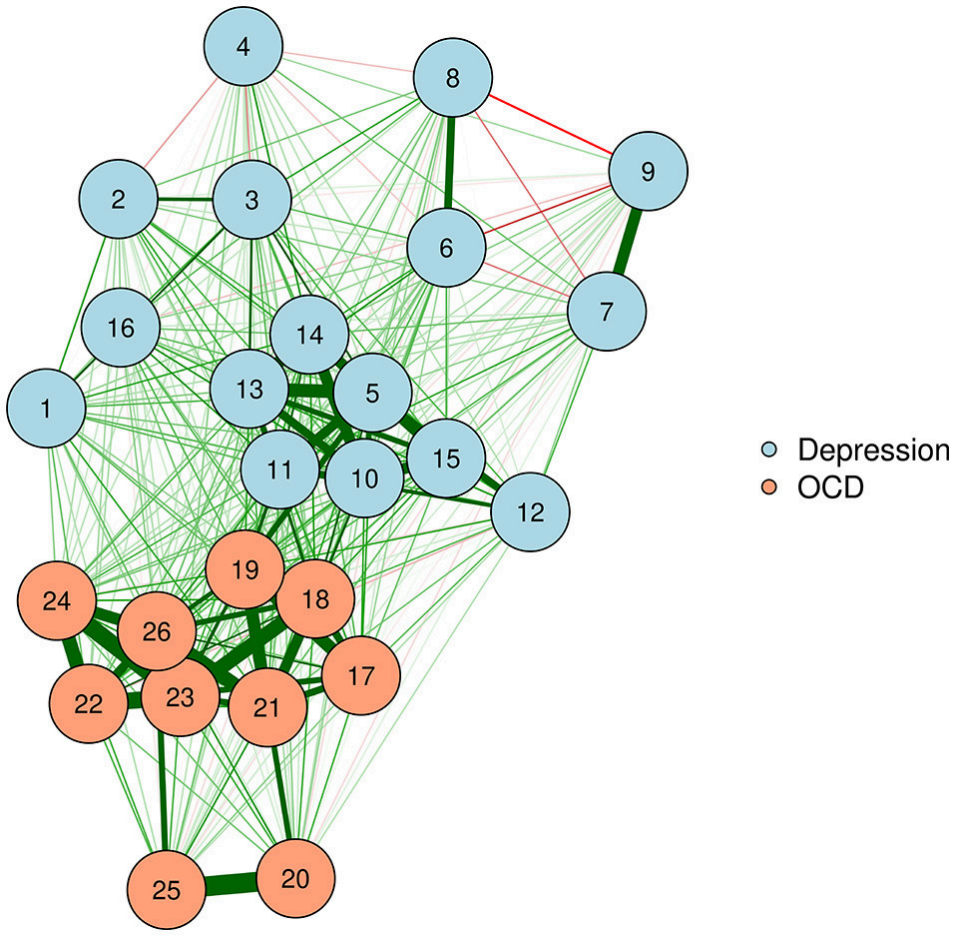
Force-directed plotting with Fruchterman–Reingold.

The FR algorithm is a default plotting method in the *qgraph* R package (Epskamp et al., 2012), and is thus very easy to implement. We will demonstrate by using a zero-order correlation network of adults with OCD and depression. The resultant network appears in Figure 1.


library("qgraph")
adult_zeroorder <- cor(Rogers)
qgraph(adult_zeroorder, **layout="spring"**,
       groups = list(Depression = 1:16,
                    "OCD" = 17:26),
                    color = c("lightblue",
                    "lightsalmon"))


Force-directed algorithms produce visually appealing plots in which nodes rarely overlap. It is important to keep in mind that the positioning of nodes in a force-directed algorithm cannot be interpreted.

## Multidimensional scaling of networks

Multidimensional scaling (MDS) has a long history and has been applied in a wide variety of academic arenas (Torgerson, 1958; Kruskal, 1964; Borg and Groenen, 2005; Borg et al., 2018). MDS represents *proximities* among variables as distances between points in a low-dimensional space (e.g., two or three dimensions; Mair et al., 2016). Proximity is an umbrella term for “similarities” between variables (e.g., correlation) or “dissimilarities” (e.g., Euclidean distance). Because MDS helps represent complex data in low-dimensional space, it dovetails precisely with the goal of visual presentation of complex psychological networks. That is, we can use MDS to represent proximities in a two-dimensional space (e.g., X & Y) to produce two-dimensional network plots. MDS is particularly useful for understanding networks because the distances between plotted nodes are interpretable as Euclidean distances. That is, highly related nodes will appear close together, whereas weakly related ones will appear far apart.

In MDS, we consider a matrix of proximities between objects (in our case, nodes). The input data for MDS can be either *directly observed proximities* or *derived proximities* (for details see Mair et al., 2016). Most psychometric networks provide us with a ready-made matrix of *derived proximities* (in this case, *similarities*): the network edges. Network edges are usually zero-order or partial correlations between pairs of nodes. Here, we will again use a zero-order correlation network as our weights matrix.


adult_zeroorder <- cor(Rogers)


Because the *smacof* R package (De Leeuw and Mair, 2009) requires dissimilarities (rather than similarities) as input, we will convert the correlation matrix into a dissimilarity matrix (Gower and Legendre, 1986; see the Data Sheet 1 (Appendix) for a formula). The result is a symmetric dissimilarity matrix Δ with *n(n-1)/2* dissimilarities (in the lower diagonal portion).


library("smacof")
dissimilarity_adult <-
    sim2diss(adult_zeroorder)


After determining our dissimilarity matrix, we then locate points (configuration matrix) in a two-dimensional space such that the distances between the objects (nodes) approximate a transformation of the dissimilarities as closely as possible, given the constraints of a two-dimensional solution. The configuration matrix for this specific application will be a matrix *X* of dimension *n x 2* with elements that represent Cartesian coordinate points with which to plot the nodes. The MDS configuration matrix provides the basis for visualization, not for any network calculations. Although in this tutorial we always constrain the configuration matrix to two dimensions (for two-dimensional plots), it should be noted that MDS can also be used to generate configurations in higher dimensions.


adult_MDS <- mds(dissimilarity_adult)
head(round(adult_MDS$conf, 2)) # top of
# configuration matrix
     D1      D2
1 −0.21    0.53
2 −0.80    0.03
3 −0.70    0.33
4  0.25   −0.77
5 −0.53   −0.07
6  0.07    0.78


### Transformations

The configuration matrix is fit on a transformation of the input dissimilarity matrix. There are several different types of transformations available. It is useful to have a variety of options for transformation so that we can choose a transformation which fits our network data. Some common transformation functions include ordinal MDS, interval MDS, ratio MDS, and spline MDS. Ordinal MDS uses a monotone step function. Ratio MDS uses a linear regression with an intercept of 0. Interval MDS is also linear but allows the intercept to vary. Spline MDS uses a monotone integrated spline. These transformations are described in greater detail in Mair et al. (2016).

**Figure 2:**
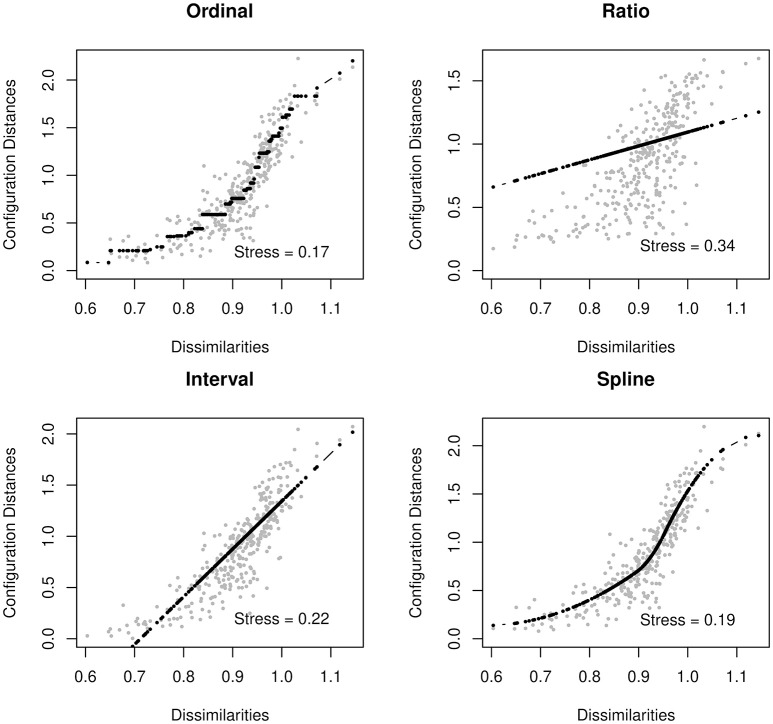
Shepard Diagrams.

In the case of psychometric networks, where we can reasonably assume that there is some metric information in the proximities, we can choose the transformation from a data-driven perspective. As with fitting any distribution, one should choose a transformation function which is both parsimonious and provides a good fit to the data. Ordinal MDS usually provides the best goodness-of-fit, but is the least parsimonious. In contrast, ratio MDS is parsimonious, but may fit poorly to some networks. We can use Shepard diagrams (Figure 2) to visualize MDS fit and to determine the preferred transformation function (Mair et al., 2016).


adult_MDS_ordinal <- mds(dissimilarity_adult,
  type="ordinal")
plot(adult_MDS_ordinal, plot.type = "Shepard",
  main="Ordinal")
text(1.1,0.3, paste("Stress =",
  round(adult_MDS_ordinal$stress,2)))



adult_MDS_ratio <- mds(dissimilarity_adult,
  type="ratio")
plot(adult_MDS_ratio, plot.type = "Shepard",
  main="Ratio")
text(1.1,0.3, paste("Stress =",
  round(adult_MDS_ratio$stress,2)))



adult_MDS_interval <- mds(dissimilarity_adult,
  type="interval")
plot(adult_MDS_interval, plot.type = "Shepard",
  main="Interval")
text(1.1,0.3, paste("Stress =",
  round(adult_MDS_interval$stress,2)))



adult_MDS_mspline <- mds(dissimilarity_adult,
  type="mspline")
plot(adult_MDS_mspline, plot.type = "Shepard",
  main="Spline")
text(1.1,0.3, paste("Stress =",
  round(adult_MDS_mspline$stress,2)))


Shepard diagrams allow us to visualize how well our MDS configuration fits our dissimilarity matrix. When the dissimilarities align in a linear fashion, a ratio or interval MDS is most appropriate. In other cases, a nonlinear transformation such as ordinal MDS or spline MDS may be more appropriate. In this case, we decided to use a spline MDS. The normalized stress values (plotted in each graph) can help guide us in deciding which transformation provides the best fit.

A value known as *stress* indicates how well one's data can be represented in two-dimensions [see Data Sheet 1 (Appendix)]. In this tutorial, we will use the *stress-1*, which is a normalized version of stress. When the stress is low, the graph is interpretable. That is, the spacing between two nodes approximately signifies the strength of their association. When the stress is higher, we must be much more cautious about these types of interpretation. A high stress indicates that the nodes cannot be accurately spaced in just two dimensions. For additional guidance on interpreting stress, see Mair et al. (2016).


adult_MDS_mspline$stress
[1] 0.189


The final product of an MDS configuration is a two-dimensional space in which distance between nodes represents the approximate dissimilarity of nodes based on their edges. For example, in our zero-order correlation network, the distance between two nodes varies inversely with their strength of association. Hence, strongly associated nodes appear close together, while weakly associated or negatively associated nodes appear far apart.

**Figure 3:**
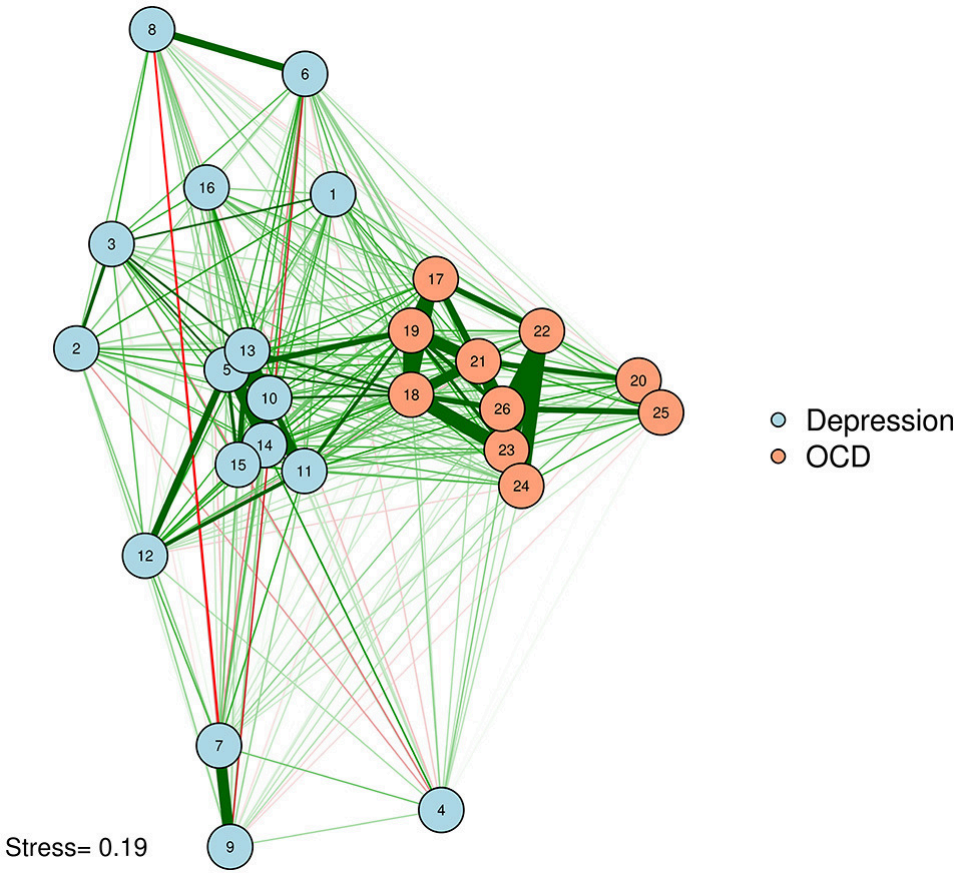
MDS configuration of a zero-order correlation network.

We can produce such a plot by entering the MDS configuration into the “layout” argument of *qgraph* or *plot*.*igraph* (Csardi and Nepusz, 2006; Epskamp et al., 2012). We will also put the stress-1 value as text on the plot, for easy reference. The result appears in Figure 3.


qgraph(adult_zeroorder,
        **layout=adult_MDS_mspline$conf**,
        groups = list(Depression = 1:16,
        "OCD" = 17:26), color = c("lightblue",
                "lightsalmon"), vsize=4)
text(-1,-1, paste("Stress=",
        round(adult_MDS_mspline$stress,2)))


McNally et al. (2017) examined a network of OCD and depression symptoms in adults. Here, a zero-order correlation network of symptoms is graphed according to a spline MDS configuration. The distance between nodes represents how close they are in terms of the zero-order correlations.

**Figure 4:**
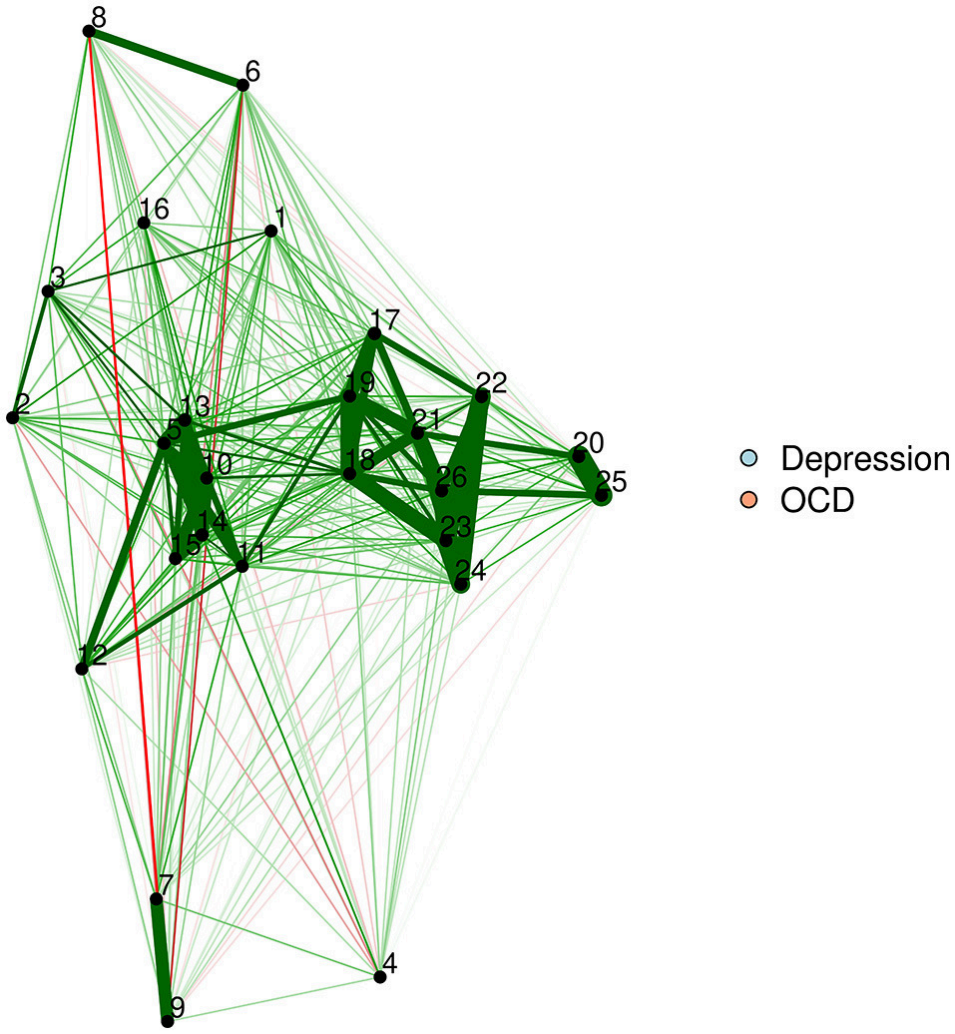
MDS configuration of a zero-order correlation network, with nodes plotted as points.

One problem with Figure 3 is that some of the strongly associated nodes overlap, obscuring the edges between those two nodes. Researchers concerned about overlap obscuring important information can reduce the size of the nodes or use points instead of circles to represent variables. Let's produce a plot with points (instead of circles) for nodes. We will use the *textplot* function in the *wordcloud* R package (Fellows, 2014) to ensure that node labels do not overlap (See Figure 4).


library("wordcloud")
qgraph(adult_zeroorder,
        layout=adult_MDS_mspline$conf,
        groups = list(Depression = 1:16,
        "OCD" = 17:26),
        color = c("lightblue", "lightsalmon"),
        **vsize=0**, **rescale=FALSE**, **labels=FALSE**)
**points(adult_MDS_mspline$conf**, **pch=16)**
**textplot(adult_MDS_mspline$conf[,1]+.03**,
**         adult_MDS_mspline$conf[,2]+.03**,
**         colnames(adult_zeroorder)**,
**         new=F)**


This figure is identical to Figure 3, but uses points to plot nodes. This avoids overlap, although some edges may remain difficult to see if the points are very close together.

Multidimensional scaling can be applied purely on the edge values in the network. This technique can be used for both psychometric networks and directly derived (e.g., social) networks. In other words, one can generate an MDS network plot based purely on the network edges, without having access to original participant data.

If one computes an MDS configuration based on the edges, the spacing between nodes is proportional to the strength of the edges. Thus, the information provided by the node spacing is redundant – represented once in the edge thickness, and yet again by the node spacing. This redundancy can facilitate quick and intuitive interpretation, but does not add new information to the plot.

**Figure 5:**
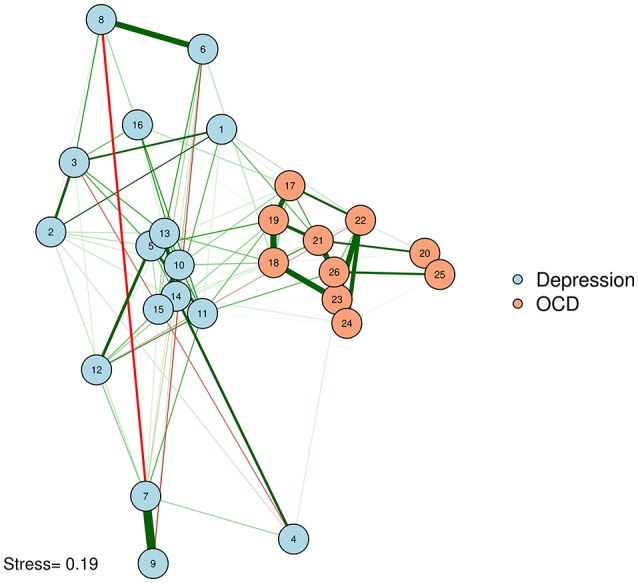
Graphical LASSO network, plotted with MDS configuration based on zero-order correlations.

If researchers want to provide *additional* information with the spacing of their nodes, they can base their MDS on a *different* type of similarity matrix derived from the original data. For example, a network could be plotted with edges that represent *partial correlations*, with spacing based on *zero-order correlations*. In other words, we could plot our partial correlation network, complete with edges, in a zero-order correlation *space*. The reverse is also possible; one could use zero-order correlations as edges, and convert a partial correlation matrix into dissimilarities as input for an MDS plotting configuration. The researcher thus maximizes the data conveyed by the graph by using the space to indicate information that is not given in the edge structure. As an example, let's compute a graphical LASSO network of the adult network, as was done by McNally et al. (2017), but use the zero-order MDS configuration from before to plot the positioning of the nodes (See Figure 5).


adult_glasso <- EBICglasso(cor(Rogers), n=408)
qgraph(**adult_glasso**,
        layout=adult_MDS_mspline$conf,
        groups = list(Depression = 1:16,
        "OCD" = 17:26),
        color = c("lightblue", "lightsalmon"),
        vsize=4)
text(-1,-1, paste("Stress=",
        round(adult_MDS_mspline$stress,2)))


Network of OCD and depression symptoms in adults (McNally et al., 2017). Here, we plot edges according to a *graphical LASSO* network, but use the graphical space between nodes to convey how closely associated nodes are in terms of the *zero-order correlations* based on an MDS configuration. In other words, nodes that are close together are similar in terms of zero-order correlations; nodes that share a thick edge are similar in terms of regularized partial correlations.

### Procrustes

**Figure 6:**
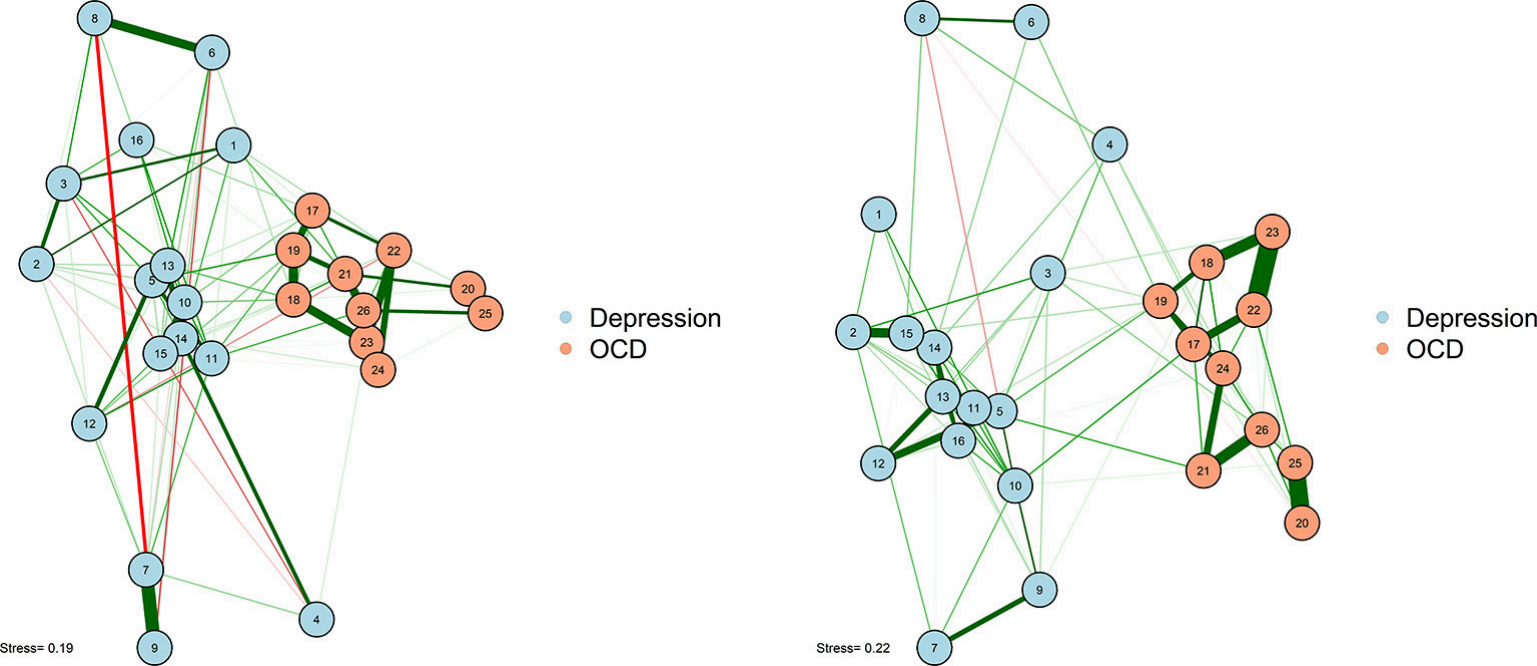
Two networks plotted using MDS configurations and Procrustes.

As noted earlier, one particularly challenging aspect of node placement is providing an accurate visual comparison between two networks. Two or more configurations can be brought into a similar space and compared by using the Procrustes algorithm [see Data Sheet 1 (Appendix); see also Davison, 1985]. This procedure, named after Poseidon's son in Greek mythology (“Procrustes, the stretcher”), removes statistically “meaningless” differences (i.e., they do not change the fit of an MDS solution) between the two configurations. We can use the Procrustes algorithm to bring together the adult network from McNally et al. (2017) with the adolescent network in Jones et al. (2018). This visual comparison is presented in Figure 6.


adolescent_zeroorder <- cor(Rogers_Adolescent)
dissimilarity_adolescent <-
  sim2diss(adolescent_zeroorder)
adolescent_MDS <- mds(dissimilarity_adolescent,
  type="mspline")
fit_procrustes <- **Procrustes(adult_MDS_**
**  mspline$conf, adolescent_MDS$conf)**
adolescent_glasso <- EBICglasso(cor
  (Rogers_Adolescent), n=87, gamma=0)
qgraph(adult_glasso, **layout=fit_procrustes$X**,
       groups = list(Depression = 1:16,
       "OCD" = 17:26),
color = c("lightblue", "lightsalmon"), title=
       "Adults, n=408", vsize=4)
text(-1,-1, paste("Stress=",
      round(adult_MDS_mspline$stress,2)))
qgraph(adolescent_glasso,
       **layout=fit_procrustes$Yhat**,
groups = list(Depression = 1:16,
         "OCD" = 17:26),
         color = c("lightblue", "lightsalmon"),
         title="Adolescents, n=87", vsize=4)
text(-1,-1, paste("Stress=",
       round(adolescent_MDS$stress,2)))


This algorithm not only creates interpretable plots; it can also be statistically evaluated in terms of how well the MDS solution replicates across different samples. The Procrustes method provides a way to compare two network plots in a highly meaningful way, where the position of nodes directly corresponds to similarities or dissimilarities between the two networks. We can even quantify the degree to which the MDS replicates between the two networks by using a congruence coefficient. A congruence coefficient is a measure of the similarity of two configurations. It is similar to a correlation coefficient, but does not extract the mean, and computes a correlation about the origin (the point [0,0]), rather than the centroid (the point around which the data are centered). This results in more favorable properties than a simple correlation for determining geometric similarity (Borg and Groenen, 2005). The congruence coefficient is generally very high, so users should not overemphasize the magnitude.


round(fit_procrustes$congcoef, 3)
[1] 0.930


An original graphical LASSO empirical network configuration and a replication in a distinct sample (Jones et al., 2017) are presented with MDS-configured networks on the zero-order correlation structures with a Procrustes transformation.

## Principal components and eigenmodels

A potentially useful alternative approach is to plot nodes within a coordinate system based on two extracted dimensions. MDS is possibly the most useful method when one wishes to meaningfully interpret the distances between nodes. In contrast, using a coordinate system provides information on how each node scores on an X criterion and a Y criterion. In a coordinate system, nodes are interpretable in terms of their “X distance” and “Y distance” from one another, but cannot be meaningfully interpreted in terms of their Euclidean distance from one another (i.e., the distance if one drew a straight line between nodes).

In principal components plotting and eigenmodels, nodes are plotted by their loadings on extracted dimensions. To be clear, these extracted dimensions do not represent latent causes. Rather, they represent aggregations of variance in the data. In some select cases, the underlying dimensions are interpretable, making the absolute position of nodes meaningful in accordance with some theoretical dimension (e.g., a dimension from physiological to nonphysiological symptoms). Because the dimensions represent aggregated variance in the data, plotting according to extracted dimensions may be useful for visualization, even if the dimensions themselves are not interpretable. Thus, a researcher may be theoretically opposed to the idea of latent dimensions as causal mechanisms of mental disorders, but still use a principal components or eigenmodel plotting approach to present a network or compare multiple networks in an easily interpretable format.

It is unavoidable that information will be lost as we attempt to represent multidimensional data in two-dimensions. This limitation is true for *all* types of network plots. In our specific application of principal components analysis (PCA) and eigenmodels, information for the graph is derived from the first two components or dimensions, and information from any additional components or dimensions is ignored.

### Principal components analysis

Principal components analysis is an excellent method for extracting meaningful dimensions on which to plot nodes. PCA and its associated rotation methods will be accessible to most psychological researchers as common methods within psychology [see Data Sheet 1 (Appendix) for technical details]. Indeed, classical MDS (e.g., Torgerson, 1958) and PCA are closely related methods. PCA can be performed in two ways: using a singular value decomposition on a dataset containing *n* observations on a set of variables (centered and divided by $\sqrt{\left(\text{n}-1\right)}$, or using an eigenvalue decomposition of the covariance (or correlation) matrix. From a network perspective, standard PCA is thus limited to psychometric networks (i.e., networks based on derived proximities) and is not designed for relational input data as in social networks.

**Figure 7:**
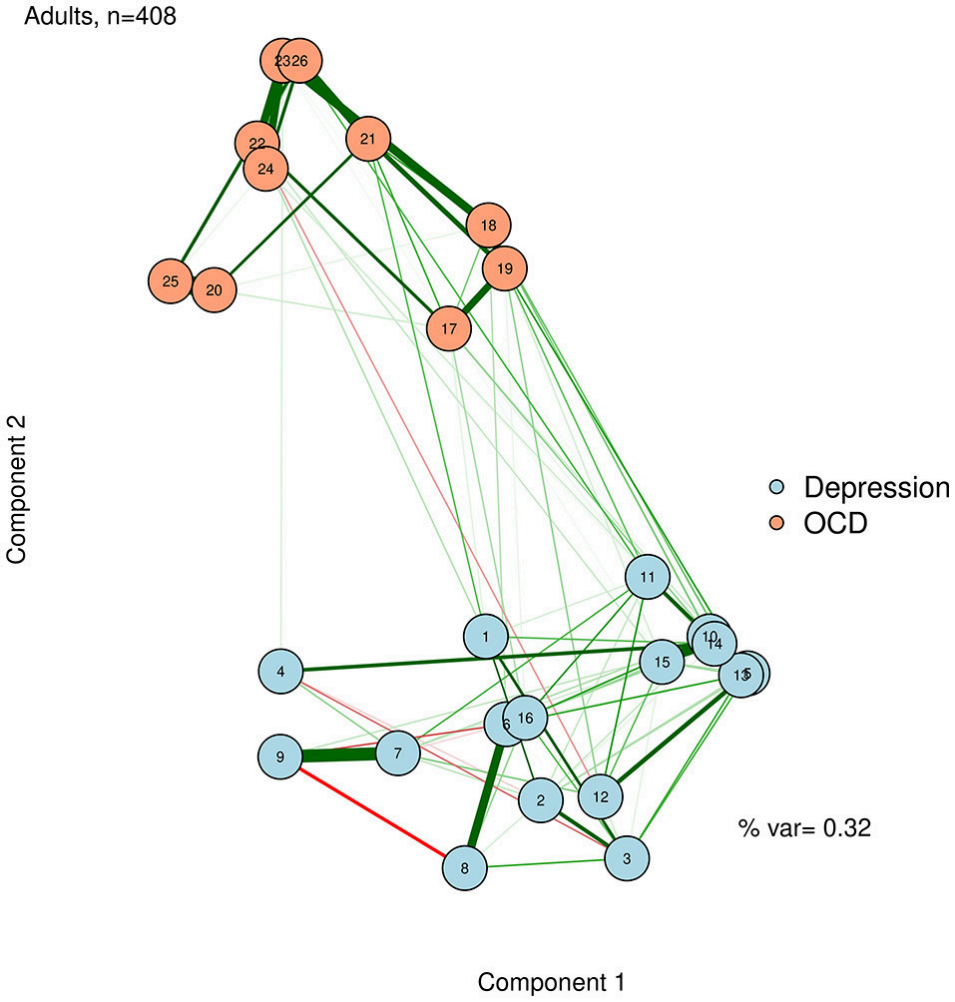
Principal components analysis configuration.

Unlike in an MDS configuration, the graphed Euclidean distance between nodes (i.e., the distance if one drew a straight line between nodes) is not meaningful in a network plotted with PCA. However, the X distance and the Y distance are *each* meaningful (e.g., how far away nodes are in horizontal space, and how far away they are in vertical space), and represent the difference between nodes on each extracted principal components. A PCA solution can be either rotated or unrotated, depending on one's preference (Joliffe, 2002). These components might or might not be meaningfully interpreted, depending on the theories regarding the network. Regardless, using the principal components as plotting mechanisms is useful to position nodes in a way that should remain largely stable across successful replications. We demonstrate this by using a varimax-rotated PCA implemented in the *psych* R package (Revelle, 2014) based on the zero-order correlation structure for the adult network (McNally et al., 2017). This visualization is presented in Figure 7.


library("psych")
PCA_adult <- principal(cor(Rogers),
                        nfactors = 2)
qgraph(adult_glasso, layout=**PCA_adult$loadings**,
groups = list(Depression = 1:16,
         "OCD" = 17:26),
         color = c("lightblue", "lightsalmon"),
         title="Adults, n=408",
         layoutOffset=c(.3,.1), vsize=4)


To facilitate interpretation, we can also add the percent variance accounted for by the first two principal components, and label the axes as “Component 1” and “Component 2.” Like the stress value in MDS, the variance accounted for by the two components can gauge how well we are capturing the complexity of the network in a two-dimensional solution. In the case of Figure 7, we accounted for a relatively low proportion of variance. Thus, even though nodes 10 and 14 are very similar in terms of the first two dimensions, we must be cautious about this interpretation, because they may differ on dimensions not captured in this plot.


text(1.5,-.8, paste("% var=",
     round(sum(PCA_adult$values[1:2]/
     length(PCA_adult$values)),2)))
title(xlab="Component 1",
     ylab= "Component 2")


The component loadings of variables (nodes) on the first two extracted dimensions from a principal components analysis can be used as the X-Y coordinates for plotting the nodes. The second component likely captures a dimension of depression vs. OCD. The first component is less clear, but after examining specific nodes, we hypothesize that it is perhaps capturing a dimension of behavioral vs. internally experienced symptoms.

### Eigenmodel networks

Eigenmodels are a type of latent variable model for symmetric relational data such as undirected networks (Hoff, 2008). They are a generalization of other popular latent variable models, such as latent class and distance models. Although eigenmodels have not yet been applied to modeling psychometric constructs, they are popular in other fields, including social network analysis (Hoff et al., 2002). Eigenmodels are extracted purely on the network structure by using a model-based eigenvalue decomposition and regression [see Data Sheet 1 (Appendix)]. The parameters are estimated through Markov chain Monte Carlo (MCMC). That is, for each parameter we extract a posterior distribution by means of which we can compute posterior means (or modes) and corresponding credibility intervals.

**Figure 8:**
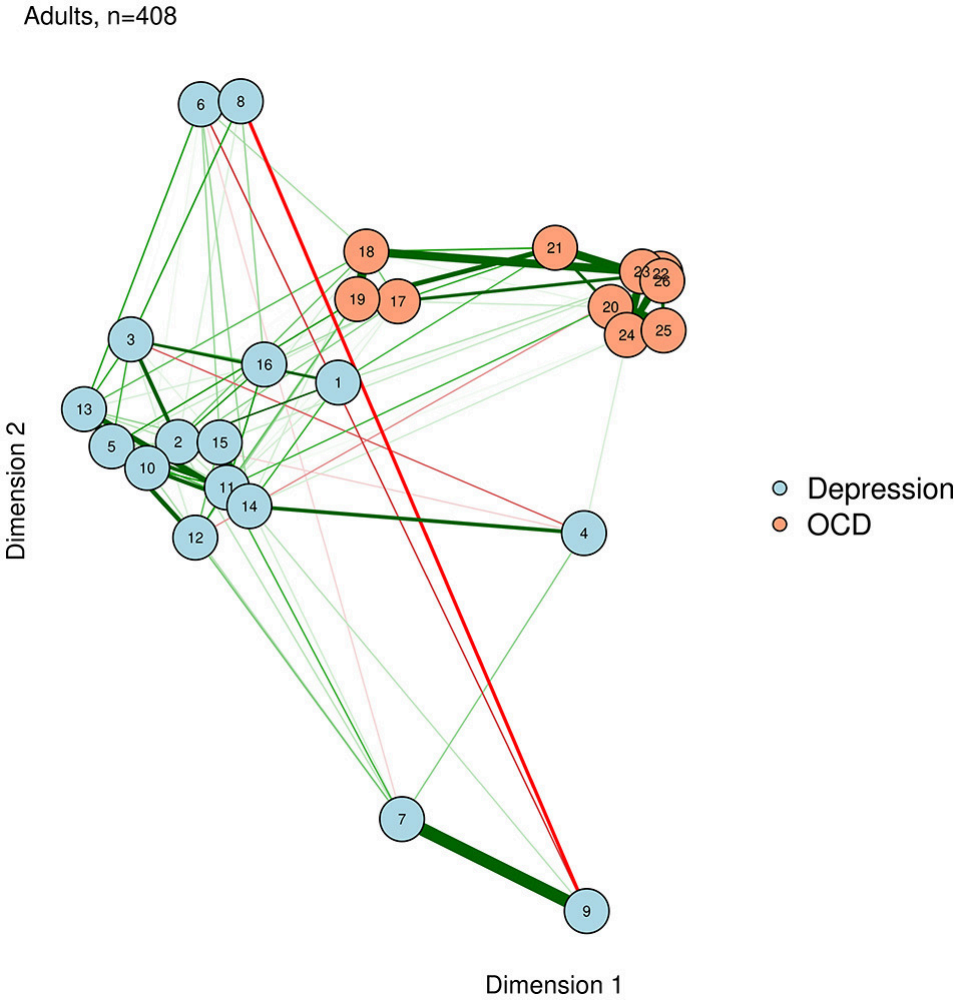
Eigenmodel configuration.

Eigenmodels allow for many interesting statistical possibilities, including attractive methods for identifying clusters (e.g., communities) of nodes. Eigenmodels also allow the researcher to study the effect of covariate variables on the structure of the weights matrix: for example, Kolaczyk and Csárdi (2014) used eigenmodels to study whether a shared office location (a plausible covariate) affected the network structure of collaborations among lawyers. Here, we emphasize that eigenmodels can provide a convenient method for the visual representation of networks in which nodes are plotted in a meaningful space. Because eigenmodels are based solely on the weights matrix (i.e., the edges), they can be computed for any network, and are not limited to psychometric networks. We demonstrate this, based on the graphical LASSO networks of the adult network, using the *eigenmodel* package (Hoff, 2012). The resultant visualization is shown in Figure 8.


library("eigenmodel")
diag(adult_glasso) <- NA ## the function
# needs NA diagonals
p <- 2 ## 2-dimensional solution
fitEM <- eigenmodel_mcmc(Y = adult_glasso,
 R = p, S = 1000, burn = 200, seed = 123)
EVD <- eigen(fitEM$ULU_postmean)
evecs <- EVD$vec[, 1:p] ## eigenvectors
# (coordinates)
qgraph(adult_glasso, layout=evecs,
       groups = list(Depression = 1:16,
       "OCD" = 17:26),
       color = c("lightblue",
       "lightsalmon"),
       title= "Adults, n=408", vsize=4)
title(xlab="Dimension 1", ylab= "Dimension
       2")


Eigenmodels extract latent dimensions directly from the weights matrix of a network. The first two dimensions determine the X and Y position of each node, respectively. For example, a node on the right side has a high loading on dimension 1, while a node near the top has a high loading on dimension 2.

## Comparing visualization methods: what to use when?

**Figure 9:**
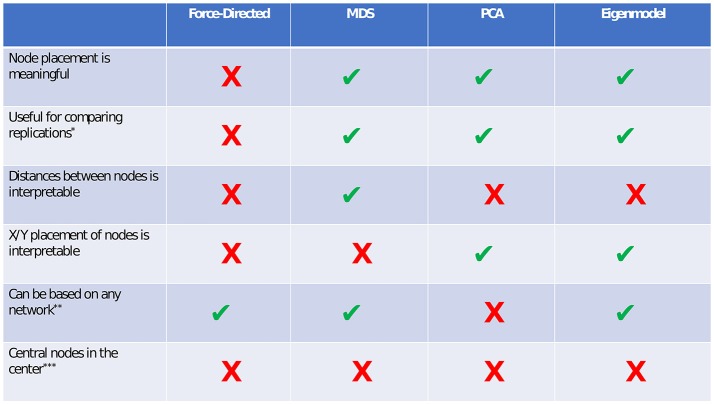
Comparison of visualization methods. *If force-directed methods are used to compare networks, the layouts should be constrained to be identical for both networks. Although this does not facilitate any spatial interpretation, it allows for easy comparison of edges. In both PCA and eigenmodels, caution should be taken in comparing networks, as the exact extracted components/dimensions will differ between datasets. **PCA relies on a correlation matrix or a set of observations. ***Although central nodes will sometimes be found near the center, we are not aware of any plotting method in which this assumption always holds.

In this tutorial, we presented four types of methods for visualizing network models: force-directed algorithms, multidimensional scaling, principal components analysis, and eigenmodels. Each of these methods has certain benefits and drawbacks. We present a summary of these costs and benefits in Figure 9.

### Force-directed algorithms

Perhaps the main benefit of force-directed algorithms is clean aesthetics. The nodes in a force-directed plot will rarely overlap, and relatively equal distance between nodes allows for easy viewing of the edges. The main drawback of force-directed methods is that the spacing between nodes is uninterpretable. This can lead to problems, especially when researchers or readers are unaware of this drawback, and make erroneous interpretations based on the node placement.

### Multidimensional scaling (MDS)

The primary benefit of multidimensional scaling is that the distances between nodes are interpretable. In other words, nodes that are close together are closely related, and nodes that are far apart are less closely related. The stress-1 value provides a helpful estimate of *how* interpretable the distances are (e.g., how well the network is reducible to two dimensions). A low stress value means that the distances are highly interpretable, and a high stress value means that the distances are not very interpretable, due to the network's high dimensionality. MDS can be used to visually compare replications of networks via the Procrustes algorithm. One drawback of MDS (compared to force-directed algorithms) is that nodes may sometimes be placed very close together, making edges harder to see. This drawback can often be alleviated by reducing the node size or by using points rather than circles to represent nodes.

### Principal components analysis (PCA)

The primary benefit of principal components analysis plotting is that the placement of nodes on the X and Y axes becomes interpretable. In other words, nodes that are far to the right differ in some dimension (i.e., component), compared to nodes on the left. The percent of variance accounted for by two components provides a helpful estimate of how interpretable the node positions are. PCA relies on a correlation matrix or a set of variable observations. Thus, one possible drawback of principal components analysis is that it specifically applies to psychometric networks (i.e., networks relying on a correlation matrix), but not to directly derived networks (e.g., social networks, where the data are not amenable to computing PCA). In PCA, edges may also be difficult to see if nodes score very similarly on both components.

### Eigenmodels

In terms of plotting and interpreting networks, eigenmodels are similar to PCA. The X and Y placement of nodes is interpretable in terms of latent dimensions of the network. One main benefit of the eigenmodel plotting approach compared to PCA is that eigenmodels can be computed from any network structure, and do not rely on the correlation matrix.

A brief comparison of the benefits and costs of different visualizations.

## Convenience functions

We hope that this tutorial provides researchers with an understanding of the methodology and rationale for using multidimensional scaling, PCA, and eigenmodels in addition to force-directed algorithms as attractive visualization methods in network analysis. In addition to using these methods as explained in the R code provided above, we have created convenience functions for these plotting methods, which facilitate ease of use at the expense of some flexibility (Jones, 2017).


library("networktools")
adult_glasso <- EBICglasso(cor(Rogers),
                       n=408)
adult_qgraph <- qgraph(adult_glasso)
MDSnet(adult_qgraph, MDSadj = cor(Rogers))
PCAnet(adult_qgraph, cormat = cor(Rogers))
EIGENnet(adult_qgraph)


## Summary

Although it is difficult to represent highly complex data in two dimensions, there are a variety of well-established methods that can accomplish this goal. Although two-dimensional representations can never fully convey the true complexity that underlies high-dimensional data, they can provide interpretable visualizations. In addition, many of these methods are capable of providing reasonable and interpretable visual comparisons across networks derived from different samples. We recommend that network researchers carefully consider the benefits and costs of each method and utilize methods that best accomplish their specific aims. We also recommend that researchers explicitly state their rationale for using certain visualization methods and provide clear instructions for how to interpret these visualizations. As researchers follow these recommendations, they will be able to furnish interpretable visualizations that clearly communicate their data to others. Perhaps more importantly, researchers will avoid misinterpretations of visualized data that lead to erroneous conclusions.

## Author contributions

PJ and PM conceived of the presented idea and developed the relevant code. PJ wrote the initial draft of the manuscript. PM wrote the initial draft of the appendix. RM supervised the project. All authors participated in critical editing and revision of the manuscript.

### Conflict of interest statement

The authors declare that the research was conducted in the absence of any commercial or financial relationships that could be construed as a potential conflict of interest.
