# Supplementary material for: Visualizing Psychological Networks: A Tutorial in R
Source: Front Psychol. 2018 Sep 19;9:1742. doi: 10.3389/fpsyg.2018.01742 (PMC6156459; doi:10.3389/fpsyg.2018.01742)
Supplement: Supplementary file 1 [file Data_Sheet_1.PDF]

Multidimensional Scaling

Let $\mathbf{R}$ be an $n \times n$ correlation matrix with elements r_{ij}, subsequently converted into a *dissimilarity matrix* $\boldsymbol{\Delta}$ of the same dimension with elements δ_{ij}. Correlations can be converted into distances using $\delta_{ij} = \sqrt{1 - r_{ij}}$. MDS scales the n objects into a low-dimensional space of dimension p. That is, it aims for find a *configuration matrix* $\mathbf{X}$ of dimension $n \times p$:

$$d_{ij}(\mathbf{X}) = \sqrt{\sum_{s=1}^{p} (x_{is} - x_{js})^2},$$

with $d_{ij}(\mathbf{X})$ as the *fitted distances*. This can be achieved by minimizing the *stress* target function:

$$\sigma(\mathbf{X}) = \sum_{i<j} (\hat{d}_{ij} - d_{ij}(\mathbf{X}))^2 \rightarrow \text{min!}$$

It uses a transformed version of the input dissimilarities $\hat{d}_{ij} = f(\delta_{ij})$. Popular transformation functions $f(\cdot)$ used in MDS are a linear transformation (*interval MDS*, or, if the intercept is omitted, *ratio MDS*), or a monotone step function (*ordinal MDS*, sometimes also called *nonmetric MDS*). The resulting transformed dissimilarities $\hat{d}_{ij}$ are called *disparities* or *d-hats*.

Various normalizations of the raw stress $\sigma(\mathbf{X})$ have been proposed in the literature. The most popular one is the *stress-1*:

$$\sigma_1(\mathbf{X}) = \sqrt{\frac{\sigma(\mathbf{X})}{\sum_{i<j} \hat{d}_{ij}^2}}.$$

Having two MDS solutions with configuration matrices $\mathbf{X}$ and $\mathbf{Y}$ both of dimension $n \times p$, they can be aligned using *Procrustes*. This procedure, named after Poseidon's son in Greek mythology ("Procrustes, the stretcher"), removes statistically "meaningless" differences (i.e., they do not change the fit of an MDS solution) between the two MDS configurations. Corresponding transformations are rotation, dilation, and translation. The steps involved in Procrustes are the following. Let $\mathbf{X}$ be the target configuration, $\mathbf{Y}$ the testee configuration to be transformed, and $\mathbf{Z}$ be a centering matrix ($\mathbf{Z} = \mathbf{I} - n^{-1}\mathbf{1}\mathbf{1}'$):

1. Compute $\mathbf{C} = \mathbf{X}'\mathbf{Z}\mathbf{Y}$.
2. SVD on $\mathbf{C}$: $\mathbf{C} = \mathbf{P}\boldsymbol{\Phi}\mathbf{Q}'$.
   - rotation matrix: $\mathbf{T} = \mathbf{Q}\mathbf{P}'$,
   - dilation factor: $s = tr(\mathbf{X}'\mathbf{Z}\mathbf{Y}\mathbf{T})/tr(\mathbf{Y}'\mathbf{Z}\mathbf{Y})$,
   - translation vector $\mathbf{t} = n^{-1}(\mathbf{X} - s\mathbf{Y}\mathbf{T}')\mathbf{1}$.
3. Final solution: $\hat{\mathbf{Y}} = s\mathbf{Y}\mathbf{T} + \mathbf{1}\mathbf{t}'$.

The resulting Procrustes configuration $\hat{\mathbf{Y}}$ can be plotted into the same MDS space as $\mathbf{X}$, subject to visual inspection of configuration differences.

Principal Component Analysis

Principal component analysis (PCA) is a procedure often used for dimension reduction that converts a set of observations of possibly correlated variables into a set of new orthogonal variables, called *principal components*. PCA is defined such that the first extracted principal component contains the largest amount of variance, and each subsequent principal component contains less variance than the last. PCA is especially useful when much of the variance in the data can be captured in just a few principal components, aiding interpretation or visualization.

Let $\mathbf{X}$ be the column-centered version of the $n \times m$ data matrix, divided by $\sqrt{n-1}$. An SVD decomposes $\mathbf{X}$ into the following three parts:

$$\mathbf{X} = \mathbf{UDV}'.$$

$\mathbf{U}$ is an $n \times m$ matrix containing the *left singular vectors*, $\mathbf{V}$ is an $m \times m$ matrix containing the *right singular vectors*, and $\mathbf{D}$ is a $m \times m$ diagonal matrix with the *singular values* on the diagonal.

The singular values reflect the standard deviations of the principal components, from which we can compute the proportion of variance explained in p dimensions. The matrix $\mathbf{U}$ contains the loadings, and the *principal component scores* can be obtained by $\mathbf{UD}\sqrt{n-1}$. Note that a PCA can also be computed via an *eigenvalue decomposition*.

Eigenmodels

The data to be considered in an eigenmodel consist of an $n \times n$ adjacency matrix $\mathbf{Y}$ (e.g., a correlation matrix), with elements Y_{ij}. In the eigenmodel, Y_{ij} is defined as a function of latent variables $\mathbf{u}_i$ and $\mathbf{u}_j$ and, optionally, a regression model with adjacency predictor vector $\mathbf{x}_{ij}$. The number of dimensions p needs to be fixed a priori.

$$Y_{ij} = f(\boldsymbol{\beta}'\mathbf{x}_{ij} + \mathbf{u}_i \boldsymbol{\Lambda} \mathbf{u}_j).$$

They eigenmodel approach uses *Markov chain Monte Carlo* (MCMC) to solve this problem. Without considering predictors, as in our example, we get the matrix $\hat{\boldsymbol{\Lambda}}$ of dimension $p \times p$ which gives the relative importance of each dimension, and the matrix $\hat{\mathbf{U}}$ of dimension $n \times p$. In this matrix, each node i gets a row vector $\hat{\mathbf{u}}_i = (\hat{u}_{i1}, \ldots, \hat{u}_{ip})$ containing the unobserved node characteristics. Nodes with similar characteristics will get similar $\hat{\mathbf{u}}$-vectors.

There is one more tweak to be applied on this solution. The vectors $\hat{\mathbf{u}}_i$ are not orthogonal, a property which is important for interpretation and plotting. Applying an eigenvalue decomposition on the fitted matrix $\hat{\mathbf{U}}\hat{\boldsymbol{\Lambda}}\hat{\mathbf{U}}'$ does the trick. The resulting eigenvectors reflect the coordinates in the p-dimensional space, and can be subject to plotting.
